# RACIAL DIFFERENCES IN PREDICTIVE CAPACITY OF EDUCATIONAL POLYGENIC SCORES ON PHYSICAL LIMITATIONS FOR OLDER ADULTS

**DOI:** 10.1093/geroni/igae098.3701

**Published:** 2024-12-31

**Authors:** Wade Catt, Micah Williams, Kenzie Latham-Mintus

**Affiliations:** Indiana University School of Medicine, Indianapolis, Indiana, United States; Indiana University School of Medicine, Indianapolis, Indiana, United States; Indiana University Indianapolis, Indiana University Indianapolis, Indiana, United States

## Abstract

This research examined whether educational polygenic scores were associated with physical limitations among older adults with European or African ancestry. In the European ancestry sample, we found that education polygenic scores were significantly associated with physical limitations in the age- and sex-adjusted models; however, education polygenic scores were no longer associated with physical limitations after adjusting for current socioeconomic status and health risk factors. In the African ancestry sample, education polygenic scores were not associated with physical limitations. Observed educational attainment was a robust predictor of physical limitations in both samples. This research demonstrates the racial inequalities in the predictive capacity of educational polygenic scores. We hypothesize that this disparity is a result of structural barriers to educational attainment by race and/or racial inequities in data collection. Both explanations stem from structural racism and highlight the limited usefulness of polygenic scores for clinical decision-making. We use these findings to explore competing theoretical explanatory frameworks of the utility and limitations of the use of polygenic scores in social and health sciences.

